# Postoperative Rehabilitation After Thyroidectomy: A Scoping Review of Stretching, Manual Therapy, and Kinesio Taping Interventions

**DOI:** 10.3390/jcm15010132

**Published:** 2025-12-24

**Authors:** Karolina Krakowska, Marcin Barczyński, Aleksander Konturek

**Affiliations:** Department of Endocrine Surgery, Jagiellonian University Medical College, 31-501 Kraków, Poland; marcin.barczynski@uj.edu.pl (M.B.); aleksander.konturek@uj.edu.pl (A.K.)

**Keywords:** thyroid surgery, postoperative rehabilitation, stretching, manual therapy, kinesio taping

## Abstract

**Background/Objectives**: Thyroidectomy is a common endocrine procedure associated with postoperative musculoskeletal symptoms such as neck stiffness, pain, and reduced cervical mobility. These sequelae, though often underrecognized, can impair recovery and quality of life. Rehabilitation strategies, including stretching, manual therapy, and kinesio taping, have emerged as potential adjuncts to enhance postoperative outcomes. This scoping review aimed to map and synthesize current evidence on postoperative rehabilitation interventions following thyroidectomy, focusing on stretching exercises, manual therapy, and kinesio taping. **Methods**: Following the Joanna Briggs Institute methodology and PRISMA-ScR guidelines, a comprehensive search identified studies evaluating physical therapy interventions in adult thyroidectomy patients. Fourteen studies published between 2005 and 2025 met the inclusion criteria, encompassing randomized trials, quasi-experimental designs, and one retrospective cohort study. Interventions were delivered in early postoperative settings and included supervised or home-based programs. **Results**: Neck stretching and range-of-motion exercises consistently demonstrated benefits in pain reduction, cervical mobility, and functional recovery. These low-cost interventions were feasible for early implementation and continuation post-discharge. Evidence for kinesio taping was mixed, with some studies reporting short-term symptom relief and others showing no significant effect. Manual therapy, assessed in a single large cohort, showed promise when combined with stretching, though its independent efficacy remains unclear. **Conclusions**: Structured rehabilitation—particularly stretching and mobility exercises—may enhance recovery after thyroidectomy. Kinesio taping and manual therapy appear beneficial as adjunctive measures but require further validation. The findings underscore the need for standardized protocols and high-quality trials to optimize postoperative care and long-term outcomes.

## 1. Introduction

Thyroid surgery ranks among the most frequently performed endocrine surgeries worldwide and is indicated for both benign and malignant thyroid disorders. While the most common complications of thyroidectomy are recurrent laryngeal nerve palsy and hypoparathyroidism, patients frequently experience additional postoperative sequelae. Although generally considered safe, the procedure can lead to neck stiffness, pain, reduced cervical and shoulder mobility, and fatigue, all of which may negatively affect daily functioning and quality of life (Kim et al. 2018 [1], Takamura et al. 2005 [2], Genc et al. 2019 [3], Syam et al. 2021 [4], Miyauchi et al. 2021 [5], Wasserman et al. 2019 [6], Albazee et al. 2024 [7], Gao et al. 2025 [8]). In the early postoperative phase, many patients report sensations of choking or tightness, accompanied by pressure, discomfort in the neck and shoulders, and limited range of motion. These symptoms are believed to result from prolonged neck hyperextension during surgery and postoperative protective behaviors, as patients tend to restrict their neck movement and maintain a rigid posture to avoid pain or tension at the incision site (Takamura et al. 2005 [2]). The musculoskeletal consequences of thyroid surgery—including soft-tissue tightness, restricted range of motion (ROM), and scar-related discomfort—are often underrecognized and insufficiently managed in clinical practice. Such issues may persist for weeks or even months, delaying full recovery and return to normal activity. If left unaddressed, persistent neck and shoulder symptoms can further contribute to impaired shoulder stability and functional decline (Miyauchi et al. 2021 [5]). Over the past few years, increasing attention has been directed toward these less severe but clinically relevant outcomes, as they may significantly influence patients’ postoperative recovery and overall satisfaction. Recent research has explored a range of rehabilitation strategies, including postural and stretching exercises, postural education, myofascial release, and joint mobilization techniques, aiming to enhance recovery and functional outcomes (Ayhan et al. 2016 [9]). However, the available literature remains heterogeneous in design and quality, with variability in methodologies, sample sizes, and follow-up durations. The absence of standardized rehabilitation protocols after thyroid surgery makes it challenging to establish clear recommendations for clinical practice.

Therefore, this scoping review aimed to map and synthesize the current evidence on postoperative rehabilitation following thyroid operations, with a focus on interventions such as stretching exercises, manual therapy, and kinesio taping, and to identify key knowledge gaps and directions for future research in this emerging field.

## 2. Materials and Methods

This review was conducted and reported in accordance with the Joanna Briggs Institute (JBI) Manual for Evidence Synthesis (Aromataris et al. 2020 [10], Peters et al., 2024 [11]) and the PRISMA-ScR (Preferred Reporting Items for Systematic Reviews and Meta-Analyses extension for Scoping Reviews) guidelines (Tricco et al. 2018 [12]). Relevant elements of the PRISMA 2020 statement were also followed where applicable. No protocol for this review was registered. The completed PRISMA-ScR checklist is provided as a Supplementary File.

### 2.1. Protocol and Registration

No protocol registration was developed for this scoping review and, therefore, no registration was performed.

### 2.2. Eligibility Criteria (PCC Framework)

This scoping review aimed to map and summarize existing evidence regarding postoperative rehabilitation after thyroidectomy, including stretching exercises, manual therapy, kinesio taping, and other physiotherapeutic interventions intended to improve neck function, reduce pain, and enhance quality of life.

The Eligibility Criteria were defined according to the population-concept-context (PCC) framework recommended by the JBI methodology for scoping reviews. Studies were included if they met all PCC elements described below.

Population: The population analyzed in this review consisted of adult patients who underwent thyroid surgery (total, near-total, subtotal, partial, or hemithyroidectomy), performed for both oncological and benign indications.

Concept: The concept focused on postoperative rehabilitation interventions, including stretching exercises, manual therapy, kinesio taping, and other physical therapy approaches aimed at improving neck mobility, reducing pain, and enhancing quality of life after thyroidectomy.

Context: The context encompassed postoperative care settings in both hospital and outpatient environments, including early rehabilitation initiated during hospitalization and home-based exercise programs implemented after discharge.

After screening titles and abstracts, full-text publications were reviewed, and studies focusing on postoperative rehabilitation after thyroid surgery were considered eligible.

Inclusion Criteria

Studies were included if they met the following criteria:

(1) Involved adult patients undergoing thyroid surgery for oncological or benign indications.
(2) Assessed any form of postoperative rehabilitation or physiotherapy intervention (stretching, kinesio taping, manual therapy, or exercise program).
(3) Reported outcomes related to pain, disability, neck range of motion, quality of life, or functional recovery.
(4) Were published in English between January 2000 and March 2025.
(5) Used quantitative study designs (randomized trials, quasi-experimental studies, cohort studies, or case series).

Exclusion Criteria

Studies were excluded if they met the following criteria:

(1) Did not include postoperative interventions.
(2) Focused on non-surgical populations.
(3) Were purely technical or surgical reports without rehabilitation data.
(4) Were conference abstracts, reviews, or non-peer-reviewed materials.
(5) Involved patients operated on for head and neck cancers other than thyroid carcinoma, patients qualified for adjuvant chemotherapy and/or radiotherapy, and those who had undergone extensive head and neck resections for advanced malignancy.

Inclusion and exclusion criteria were applied during the title and abstract screening stage and subsequently reassessed during the full-text evaluation.

### 2.3. Information Sources and Search Strategy

The search was conducted across five major databases: PubMed, Scopus, Embase, Web of Science, and the Cochrane Library. The search was performed in August 2025 and was limited to studies published between January 2000 and March 2025. Searches were performed independently by two reviewers (K.K. and M.B.). The third author (A.K.) reviewed the extracted data for accuracy and completeness and manually screened reference lists of all included articles to identify additional eligible studies.

The search followed the three-step approach recommended by the JBI Manual for Evidence Synthesis (Aromataris et al. 2020 [10], Peters et al. 2024 [11]).

In the first step, a limited preliminary search of PubMed and other databases was conducted to identify relevant keywords and MeSH terms. In the second step, a comprehensive search combining keywords and index terms was adapted for each database. In the third step, reference lists of included articles were screened for additional studies. The keywords used for the search were thyroidectomy, thyroid surgery, rehabilitation, physiotherapy, kinesiotherapy, and quality of life.

The full electronic search strategy is provided in Supplementary Table S1.

### 2.4. Selection of Sources of Evidence

Duplicate records were removed manually during the initial screening. Two reviewers independently screened the titles and abstracts of all retrieved records. Full texts of potentially eligible studies were assessed independently against the predefined inclusion and exclusion criteria. Any discrepancies were resolved through discussion or, when necessary, consultation with a third reviewer. Reasons for exclusion at the full-text screening stage were predefined and are reported in the Section 3 and illustrated in the PRISMA flow diagram.

**Figure 1:**
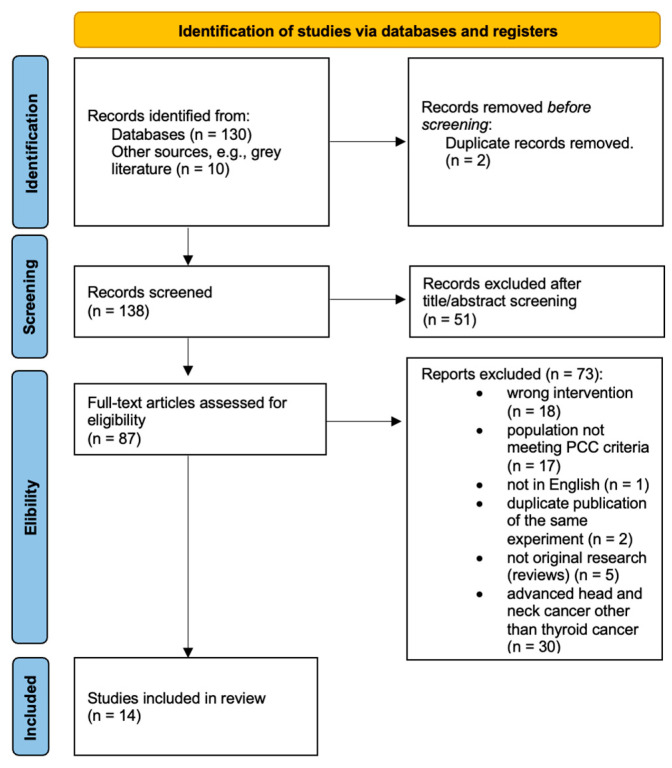
PRISMA flow diagram.

The selection process followed the PRISMA-ScR guidelines. The number of records identified, screened, and included is presented in the PRISMA flow diagram (Figure 1).

### 2.5. Data-Charting Process

Data charting was conducted using a standardized extraction form developed in accordance with the JBI Manual for Evidence Synthesis [10]. Two reviewers (K.K. and M.B.) independently charted data from each included study. Before full data extraction, the charting was pilot-tested on three studies to ensure consistency and clarity of the variables, and minor adjustments were made accordingly. For each study, the following information was extracted: authors, year of publication, country, study design, sample size, population characteristics, details of the postoperative intervention (e.g., stretching exercises, manual therapy, and kinesiotherapy), presence of any type of comparator (e.g., standard postoperative care), timing of the intervention, measured outcomes (pain, disability, range of motion, and quality of life), follow-up duration, and key findings. Discrepancies between reviewers were resolved through discussion, and when necessary, by consulting a third reviewer. Data organization and descriptive synthesis were performed using Microsoft Excel (Microsoft Corporation, Redmond, WA, USA), Microsoft 365 version.

The extracted data are summarized in Table 1 (detailed results are provided in Supplementary Table S2).

### 2.6. Critical Appraisal

The methodological quality of the included studies was assessed using the JBI Critical Appraisal Checklists [22] appropriate for each study design (randomized controlled trials, quasi-experimental studies, and cohort studies). Two reviewers (K.K. and M.B.) independently appraised each study, and any disagreements were resolved through discussion or consultation with a third reviewer (A.K.).

**Figure 2:**
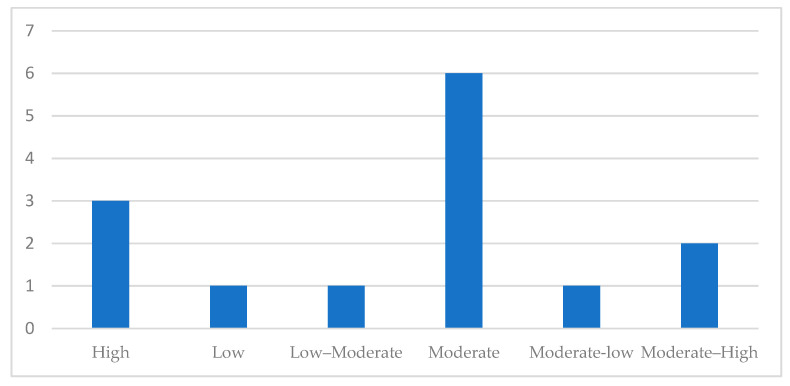
Summary of methodological quality ratings of the included studies according to the JBI Critical Appraisal tools. The figure presents the proportion of studies rated as high-, moderate-, or low-quality studies.

Each study was categorized as having high, moderate, or low methodological quality based on the proportion of ‘Yes’ responses. In accordance with JBI guidance for scoping reviews, the results of the critical appraisal did not determine study inclusion but were used to contextualize the evidence and inform the interpretation of the findings. A detailed summary of the JBI quality assessment results is provided in Supplementary Table S3 and Figure 2.

### 2.7. Synthesis of Results

Data extracted from the included studies were synthesized descriptively in accordance with the objectives of this scoping review. A narrative synthesis was undertaken to map the characteristics of the evidence, summarize intervention types, and describe outcome domains. No meta-analysis was performed, as the heterogeneity of study designs, interventions, and outcome measures precluded quantitative pooling.

The findings were organized according to study design, characteristics of the postoperative rehabilitation intervention, comparator, timing of the intervention, and outcome categories. Descriptive statistics were used to summarize the study distributions, while tables and pivot charts were used to present patterns across interventions, measurement tools, and reported outcomes. The results of the methodological appraisal were used to contextualize and interpret the strength and consistency of the evidence base.

Visual summaries of the study characteristics and outcome domains are presented in Supplementary Figures S1 and S3. Detailed descriptive data are available in Supplementary Table S3.

## 3. Results

### 3.1. Study Selection

A total of 130 records were identified through database searching, and an additional 10 records were identified through other sources, such as grey literature and manual reference screening. After removing 2 duplicates (n = 2), 138 unique records were screened based on title and abstract. Following this step, 51 records were excluded as irrelevant to the review topic. The full texts of 87 articles were assessed for eligibility, of which 73 were excluded for predefined reasons, including wrong intervention, populations not meeting the PCC criteria, non-English language, duplicate publication, non-original research, and advanced head and neck cancer. Finally, 14 studies fulfilled the inclusion criteria and were included in this scoping review. The detailed selection process is illustrated in the PRISMA flow diagram (Figure 1).

### 3.2. Study Characteristics

A total of 14 studies published between January 2005 and March 2025 were included in this review. The studies were conducted in seven countries, including China (n = 2), Denmark (n = 1), Egypt (n = 5), India (n = 1), Japan (n = 1), Korea (n = 1), and Turkey (n = 3). Most studies employed a quasi-experimental design (n = 8) or a randomized controlled trial (RCT) design (n = 5), while the remaining one was a retrospective cohort (n = 1) study.

The sample size ranged from 5 to 733 patients, with a median of approximately 75 subjects per study. All studies included adult patients after total or less than total thyroidectomy, performed for either benign or malignant thyroid disease.

The follow-up period varied from the immediate postoperative period to 12 weeks after surgery. The interventions were primarily delivered during early postoperative recovery (within 1–2 weeks after surgery) and continued as supervised or home-based exercise programs. Comparators included standard postoperative care or alternative rehabilitation protocols, depending on the study. Studies reported a range of postoperative outcomes, most commonly pain, cervical range of motion, disability, quality of life, and functional recovery. A detailed summary of all included studies, including intervention types, comparators, and outcome domains, is provided in Supplementary Table S2 (a summary of the included studies). The geographical and methodological distribution of the studies is presented in Supplementary Figure S3 (the numbers of studies by country and study design). Detailed study-level characteristics and full reference information for all included studies are provided in Supplementary Tables S2 and S3.

### 3.3. Interventions

The included studies evaluated a range of rehabilitation interventions aimed at improving neck mobility, pain control, and overall quality of life after thyroidectomy. Stretching and cervical range-of-motion (ROM) exercises were the most frequently investigated approaches, reported in thirteen studies. Across the included studies, rehabilitation interventions were initiated predominantly in the early postoperative period, most commonly during hospitalization or within the first 1–2 weeks following thyroidectomy. Intervention protocols varied substantially in frequency and duration, from brief supervised sessions during hospitalization to structured home-based programs lasting 4 to 12 weeks. Several studies implemented multimodal rehabilitation programs, combining stretching with neck and shoulder mobility training under a physiotherapist’s supervision. One study (Mao et al. 2024 [15]) examined manual therapy techniques, including soft-tissue mobilization and myofascial release as passive rehabilitation performed by a physiotherapist simultaneously with stretching exercises, and reported an improvement in quality of life and neck range of motion. Kinesio taping (KT) was examined in two studies as an intervention targeting postoperative neck pain and muscle stiffness (Nagib et al. 2019 [16], Genc et al. 2019 [3]). Nagib et al. compared KT with stretching exercises and reported that stretching resulted in a greater reduction in disability and improvement in neck range of motion compared with KT. Conversely, Genc et al. observed no significant differences between groups (KT vs. sham-KT) regarding pain intensity, range of motion, and disability.

Control groups mostly received routine postoperative care, which typically included medical management, wound care, and unsupervised self-movement without structured rehabilitation.

Across the evidence base, thirteen of fourteen included studies reported beneficial postoperative changes associated with rehabilitation interventions, particularly in pain reduction, disability scores, and cervical ROM, with several studies also demonstrating improvements in quality of life. A detailed overview of intervention types and comparators is presented in Supplementary Table S2, while the frequency of outcomes and assessment tools is summarized in Supplementary Figures S2 and S3.

### 3.4. Outcome Domains and Measurement Tools

A variety of clinical and functional outcomes were evaluated, reflecting postoperative disability, cervical mobility, and quality of life following thyroidectomy. The most frequently assessed domains were pain (11/14 studies), disability (10/14 studies), range of motion (4/14 studies), and quality of life (3/14 studies). Several studies also assessed secondary outcomes, such as shoulder function, voice-related outcomes, fatigue, anxiety, and self-efficacy.

Pain intensity was most commonly measured using the visual analog scale (VAS) or numeric rating scale (NRS). Two studies (Takamura et al. 2005 [2] and Genc et al. 2019 [3]) additionally monitored analgesic consumption, which served as an indicator of pain and postoperative recovery. Disability was evaluated primarily using the Neck Disability Index (NDI and NDII), applied in six of the fourteen included studies. Quality of life was measured using validated thyroid-specific or general instruments, such as the Thy-PRO-39, EORTC QLQ-C30, QLQ-C30, EQ-5D, and Thy-PRO-39-TOETVA. Shoulder and neck range of motion (ROM) were quantified with goniometric or inclinometric measurements or through standardized manual evaluation techniques.

Psychological or fatigue-related symptoms were assessed in selected studies using instruments such as the Hospital Anxiety and Depression Scale (HADS-A) or the Brief Fatigue Inventory (BFI). One study analyzed immune function through natural killer (NK) cell activity.

Overall, the outcome assessment was heterogeneous, reflecting the multidimensional nature of postoperative recovery after thyroid surgery. The distribution of outcome domains and the corresponding measurement tools is illustrated in Supplementary Figures S2 and S3, and detailed study-level data are provided in Supplementary Table S3.

### 3.5. Methodological Quality of Included Studies

The methodological quality of the included studies varied across designs. Using the JBI Critical Appraisal Checklists appropriate for each study type, most randomized controlled trials demonstrated moderate methodological rigor, whereas quasi-experimental and observational studies showed more substantial limitations.

Among RCTs, strengths included clearly described eligibility criteria and adequate reporting of baseline characteristics. However, several trials lacked sufficient detail on randomization procedures or allocation concealment, and blinding of participants or outcome assessors was rarely reported. The predominance of subjective outcome measures (pain ratings and disability scales) also increased susceptibility to performance and detection bias.

The quasi-experimental studies frequently relied on quasi-random allocation methods (e.g., week-of-surgery assignment or odd-even allocation). Blinding was absent in all quasi-experimental designs, and descriptions of adherence monitoring or handling of missing data were limited. Many of these studies used small samples and single-center recruitment, further limiting internal validity.

The single retrospective cohort study scored lower on several JBI criteria due to its non-randomized design, lack of control for confounding variables, and reliance on retrospective medical record extraction.

Overall, most studies were rated as moderate quality, reflecting adequate reporting of study aims and outcomes but recurring methodological weaknesses related to randomization, blinding, confounding, and follow-up completeness. A summary of appraisal outcomes is presented in Figure 2, and detailed item-level JBI assessments are provided in Supplementary Table S3.

### 3.6. Summary of Findings

Across the fourteen included studies, structured postoperative rehabilitation after thyroidectomy was generally associated with reduced neck pain, improved cervical range of motion (ROM), and lower disability scores compared with routine care or unstructured activity.

Stretching and ROM exercises, whether supervised sessions or home-based, consistently produced short-term improvements in pain and mobility, and frequently reduced disability indices. Manual therapy improvement in scar or soft-tissue flexibility in small samples, although protocols and dosing differed across studies. Evidence for kinesio taping (KT) was mixed: one study reported greater improvements with stretching than with KT, while another found no significant differences between KT and sham-KT.

Several studies reported short-term improvements in quality of life (QoL), although heterogeneity in measurement instruments and follow-up timelines limited direct comparisons.

No serious adverse events related to rehabilitation were reported.

Overall, the current evidence suggests that early, structured stretching and ROM programs, alone or combined with adjunct approaches such as manual therapy or KT, are safe and associated with beneficial short-term postoperative outcomes. However, methodological heterogeneity, small sample sizes, limited protocol standardization, and short follow-up durations constrain conclusions regarding optimal intervention methods and long-term effectiveness.

## 4. Discussion

### 4.1. Overview of Main Findings

This scoping review synthesized evidence from 14 studies evaluating postoperative rehabilitation interventions following thyroidectomy. The included evidence base consisted of five randomized controlled trials (RCTs), eight quasi-experimental studies, and one retrospective cohort study. Across these studies, the most consistent improvements were observed with neck stretching and range-of-motion (ROM) exercises, which were associated with reductions in pain intensity and functional disability, as well as improvements in cervical mobility. These effects appeared most when exercises were introduced early in the postoperative period and delivered as structured, supervised, or home-based programs during the first week after surgery.

Manual therapy was examined in a single retrospective cohort study (involving 733 patients) incorporating soft-tissue mobilization and myofascial release within a multimodal program that also included stretching exercises. Although improvements in ROM and quality of life were reported, the combined nature of the intervention prevented isolation of the independent effect of manual therapy.

The findings regarding kinesio taping (KT) were inconsistent. Two analyzed studies (Genc et al. 2019 [3], Nagib et al. 2019 [16]) reported no statistically significant advantage of kinesio taping compared with stretching exercises or sham-KT. Genc et al. observed no meaningful difference between correctly applied KT and randomly applied tapes, suggesting that placebo or sensory stimulation effects may play a role.

Overall, although study designs and intervention protocols varied widely, most evidence indicated that structured movement-based rehabilitation—particularly stretching and mobility exercises—supports postoperative recovery after thyroidectomy, while evidence for manual therapy and KT remains preliminary.

### 4.2. Interpretation of Evidence

The findings of this review highlight the importance of early, structured postoperative rehabilitation following thyroid surgery. The most reproducible benefits related to pain reduction and improvements in cervical range of motion, both of which are critical for restoring normal daily function and preventing long-term postoperative stiffness (Miyauchi et al. 2021 [5]).

Improvement in quality of life (QoL) was reported in studies that extended rehabilitation beyond the hospital stay, particularly with home-based follow-up programs lasting several weeks. This suggests that continued engagement in postoperative exercise may aid sustained functional and psychosocial recovery.

In contrast, the effects of manual therapy and kinesio taping were less consistent. Manual therapy appeared beneficial in a large retrospective study, but was implemented alongside stretching, making it difficult to isolate its independent contribution. KT showed mixed results. While some transient relief of stiffness and pain was reported, another study found no significant difference from randomly applied tape, implying that placebo or sensory effects might explain part of the observed outcomes.

The heterogeneity of study protocols, intervention dosage, and outcome measures remains a major limitation. The included studies differed widely in intervention timing, frequency, intensity, and evaluation tools, which complicates comparison and precludes identification of optimal rehabilitation parameters. Combined with small or single-center samples, short follow-up durations, and reliance on subjective outcomes, these factors hinder the development of a standardized postoperative rehabilitation protocol after thyroid surgery.

### 4.3. Methodological Considerations

The randomized controlled trials (RCTs) demonstrated moderate methodological rigor, although substantial variability existed in randomization procedures and reporting transparency. Randomization methods included computer-generated sequences (Ayhan et al. 2016 [9], Turkmen et al. 2022 [19]), random number tables (Xu et al. 2024 [13]), and block randomization by week of surgery (Thorsen et al. 2022 [14]). However, in several trials, the details of allocation concealment were either insufficiently described or absent. Only one study explicitly stated the use of a double-blind design (Genc et al. 2019 [3]), though without specifying the blinding method and, in most studies, participants and assessors were not blinded. The predominant use of subjective outcome measures (e.g., pain and self-reported disability scales) increased the risk for performance and detection bias.

The quasi-experimental studies exhibited similar methodological limitations. Several studies described some form of random allocation, yet the procedures were often quasi-randomized rather than truly random; for instance, allocation based on the week of surgery (Takamura et al. 2005 [2]) or the odd–even method (Nagib et al. 2019 [16]). Only one study (Khamis et al. 2021 [21]) explicitly stated the use of a computer-generated randomization table, although no assessor blinding was applied. Across all quasi-experimental designs, blinding of participants and outcome assessors was consistently absent, and descriptions of allocation concealment were not provided. Most studies relied on self-reported or subjective measures of neck discomfort and functional recovery with limited use of validated instruments, further contributing to potential measurement bias.

The only observational study included in this review was a retrospective cohort analysis, involving 733 participants, which provided valuable large-scale data on postoperative rehabilitation outcomes after thyroid surgery. However, the study was inherently limited by its non-randomized design and dependence on retrospective medical record data. The absence of blinding, potential selection bias, and lack of control for confounding variables further limited causal interpretation.

### 4.4. Clinical Implications

The findings of this scoping review highlight the potential value of integrating structured rehabilitation programs into the postoperative care of patients undergoing thyroid surgery. Across the included studies, neck stretching and range-of-motion (ROM) exercises were consistently associated with pain reduction, neck mobility, and functional recovery. These interventions are simple, low-cost, and feasible for both in-hospital initiation and continuation as part of a home-based exercise program, supporting their suitability for early implementation after surgery.

The results suggest that postoperative management should extend beyond standard wound care and routine follow-up, including guided mobility training to prevent long-term stiffness and improve overall comfort.

Early initiation of gentle stretching—once wound stability allows—may facilitate tissue adaptation and enhance recovery of neck function.

In contrast, the evidence regarding kinesio taping (KT) remains inconclusive. Some studies reported short-term pain relief and improved neck comfort, while others found no significant difference compared with standard care or randomly applied tape (Nagib et al. 2019 [16], Genc et al. 2019 [3]). Given these inconsistent results, KT should currently be considered an optional adjunctive measure rather than a core component of postoperative rehabilitation.

Manual therapy, evaluated in a single large retrospective cohort, appeared to contribute to improved outcomes when combined with stretching exercises. However, the multimodal nature of that intervention prevents firm conclusions about its independent efficacy.

Overall, the collective evidence supports the clinical relevance of early, structured movement-based rehabilitation, yet underscores the need for standardized protocols and methodologically rigorous trials to establish optimal timing, frequency, and intensity of exercises. Incorporating individualized rehabilitation plans into thyroidectomy care pathways may improve both physical recovery and patient-reported quality of life, aligning with broader trends toward multidisciplinary, function-oriented postoperative management.

### 4.5. Limitations and Future Directions

This scoping review has several limitations that should be acknowledged.

Although a comprehensive search strategy was applied across multiple databases and grey literature sources, only 14 studies met the inclusion criteria, many of which were small, single-center investigations with limited generalizability. Considerable heterogeneity of study designs, intervention protocols, and outcome measures precluded quantitative synthesis and prevented formal meta-analysis. Most included studies originated from specialized surgical centers, which may restrict applicability to community or primary care settings. In addition, variations in surgical techniques and postoperative management protocols across studies may have influenced outcomes independently of rehabilitation interventions.

Methodological limitations were common across the evidence base. These included incomplete reporting of randomization methods, lack of blinding, reliance on subjective outcome measures, and short follow-up durations, all of which constrained interpretation of long-term effectiveness. The predominance of self-reported tools (e.g., pain intensity or neck disability) further increases the likelihood of measurement bias. The single retrospective cohort study, despite its large sample, was limited by the inherent constraints of its observational design, including potential selection bias and uncontrolled confounding.

From the perspective of this review’s methodology, the evidence was drawn primarily from studies published in English, but it also incorporated several papers from grey literature sources, particularly journals with regional circulation. The included studies originated mainly from Egypt, Turkey, China, South Korea, and India, with one study from Denmark, reflecting a diverse but regionally concentrated research landscape. Although this enhances the inclusiveness of this review, it may also limit the generalizability of findings to Western or high-income clinical contexts, where postoperative rehabilitation practices and healthcare infrastructure may differ.

As a scoping review, this study aimed to map the existing evidence and did not appraise study quality for weighting or excluding studies from the synthesis, in accordance with the PRISMA-ScR methodology. This approach enhances comprehensiveness but limits the ability to draw firm comparative conclusions.

Future research should focus on well-designed, adequately powered randomized controlled trials using standardized rehabilitation protocols and validated, objective outcome measures.

Comparative studies evaluating different modalities of physical therapy, such as stretching, manual therapy, and adjunctive techniques such as KT, are particularly needed to establish evidence-based guidelines.

Additionally, long-term follow-up is essential to assess the persistence of functional improvements and quality of life benefits.

A multidisciplinary approach involving surgeons, physiotherapists, and rehabilitation specialists could facilitate the development of structured care pathways to optimize postoperative recovery after thyroidectomy.

## 5. Conclusions

This scoping review mapped the existing evidence on postoperative rehabilitation after thyroid surgery and identified a growing interest in structured movement-based interventions as part of postoperative care. The most consistent benefits were associated with neck stretching and range-of-motion exercises, which were linked to reductions in pain and improvements in cervical mobility and quality of life. Evidence regarding kinesio taping and manual therapy remains limited and inconsistent, suggesting that these modalities should currently be considered adjunctive rather than primary interventions.

Although the methodological quality of the included studies varied, the overall direction of findings supports the integration of early, guided rehabilitation into standard postoperative management following thyroid surgery.

Future well-designed, adequately powered trials using standardized protocols and validated outcome measures are needed to establish evidence-based protocols and to clarify the long-term functional benefits of postoperative rehabilitation in this patient population.

## Figures and Tables

**Table 1:** Summary of included studies. The full extraction dataset can be found in the Supplementary Materials (Table S2).

Study (Year)	Sample Size, *n*	Study Design	Intervention	Main Results	Adverse Effects
Xu et al. (2024) [13]	409	Randomized controlled trial	Shoulder and neck exercises + standard care vs. standard care	Both groups improved over time. The intervention group showed significantly better pain and total scores at 1 day, 1 week, and 1 month (*p* < 0.05). No between-group differences in ADL, ROM, or muscle strength were found; the intervention group showed higher self-efficacy, emotional, cognitive, and overall health scores, and lower pain scores (*p* < 0.05).	None mentioned
Genc A. et al. (2019) [3]	89	Randomized, double-blind, sham-controlled study	Kinesio taping vs. sham-kinesio taping	No between-group differences in ROM, neck pain, or disability were observed; analgesic use significantly reduced.	Skin hypersensitivity in one participant
Takamura Y. et al. (2005) [2]	733	Prospective, single-center, quasi-randomized study	Stretching exercises vs. no intervention	Stretching group showed significantly lower neck symptom scores and NSAID use at all time points.	None mentioned
Thorsen R.T. et al. (2022) [14]	80	Single-blinded randomized controlled trial	Stretching exercises vs. no intervention	No between-group differences in thyroid-specific QoL, voice function, neck/shoulder pain, or general health were observed.	No important harm or unintended effects were observed
Mao Y. et al. (2024) [15]	82	Retrospective cohort study	New rehabilitation training (active training + passive rehabilitation-myofascial release) vs. conventional rehabilitation training (functional exercises performed by patients themselves)	QoL was significantly higher and recovery better in the intervention group; cervical ROM was also significantly better at 1 and 3 months.	None mentioned
Kim K. (2018) [1]	62	Quasi-experimental study with a non-equivalent control group	Home-based exercise program (aerobic training, such as walking, resistance exercises, and flexibility exercises) vs. no intervention	Experimental group had significantly less fatigue and anxiety, higher QoL, and greater NK cell activity (*p* < 0.05).	None mentioned
Ayhan H. et al. (2016) [9]	43	Randomized controlled trial	Stretching exercises vs. no intervention	Stretching group had significantly less pain, disability, and neck sensitivity at 1 week; no differences between groups were observed after 1 month.	None mentioned
Nagib S.H. et al. (2019) [16]	90	Quasi-experimental pre-test–post-test study	Stretching exercises vs. kinesio taping for posterior neck muscles	Neck ROM significantly improved in both groups, with greater post-treatment gains in the stretching group; no between-group difference in NPDS were observed.	None mentioned
Bhavani D.E. et al. (2018) [17]	5	Quasi-experimental one-group pre-test–post-test study	Stretching exercises	Education and neck stretching significantly reduced neck pain and disability.	None mentioned
Zeinab M.A.M. et al. (2019) [18]	30	Quasi-experimental study with control group	Stretching exercises vs. no intervention	Pain and disability levels were significantly lower in the intervention group at 1 week and 1 month.	None mentioned
Turkmen A. et al. (2022) [19]	76	Randomized controlled trial	Head and neck exercises vs. no intervention	No between-group difference in NPDS at 1 week or 1 month was observed; both groups showed significant improvement in neck pain and discomfort over time.	None mentioned
Abo Shehata OK et al. 2020 [20]	50	Quasi-experimental study with control group	Neck exercises (range of motion) vs. no intervention	Pain and disability were significantly lower in the intervention group at 1 week and 1 month.	None mentioned
Khamis E.A. et al. (2021) [21]	60	Quasi-experimental, comparative interventional study	Active range of motion (Active ROM): independent neck and shoulder exercises without assistance vs. self range of motion (Self ROM): assisted neck and shoulder exercises using the contralateral arm	Self-ROM group showed higher muscle strength, better ROM, and lower shoulder pain at 1 month and better DASH and NDII scores at 1–2 months.	None mentioned
Syam N. et al. (2021) [4]	74	Quasi-experimental study with control group	Head and neck stretching exercises (+routine hospital care) vs. no intervention (routine hospital care)	At 1 and 4 weeks, the intervention group showed significantly lower pain and disability (*p* < 0.001), with pain improving from severe to mild and greater functional gains across daily activities.	None mentioned

## Data Availability

All data included in this study are available in the published articles analyzed in this review. No new data were created or analyzed in this study.

## Supplementary Materials

The following supporting information can be downloaded at: https://www.mdpi.com/article/10.3390/jcm15010132/s1, Table S1: Full electronic search strategy for all databases (PubMed, Scopus, Embase, Web of Science, and Cochrane Library). Table S2: Extended summary of included studies (full extraction table with detailed study characteristics, intervention descriptions, and outcome domains). Table S3: Full comparison of included studies, summarizing key methodological characteristics, interventions, outcome domains, main findings, and JBI Critical Appraisal rating for each study. Figure S1: Distribution of studies by country (pivot chart illustrates the geographical origin of all studies included in this scoping review). Figure S2: Frequency of reported measurement tools across included studies (pivot chart summarizes the distribution of clinical and patient-reported outcome instruments applied to evaluate postoperative recovery domains, such as pain, disability, cervical mobility, and quality of life). Figure S3: Frequency of reported outcome domains across included studies (pivot chart shows the frequency with which pain, disability, cervical range of motion, quality of life, and other postoperative recovery domains were evaluated); File S1: PRISMA-ScR Checklist.
