# Supplementary material for: Postoperative Rehabilitation After Thyroidectomy: A Scoping Review of Stretching, Manual Therapy, and Kinesio Taping Interventions
Source: J Clin Med. 2025 Dec 24;15(1):132. doi: 10.3390/jcm15010132 (PMC12787179; doi:10.3390/jcm15010132)
Supplement: Supplementary file 1 [file jcm-15-00132-s001.zip › Supplementary Figure S3.pdf]

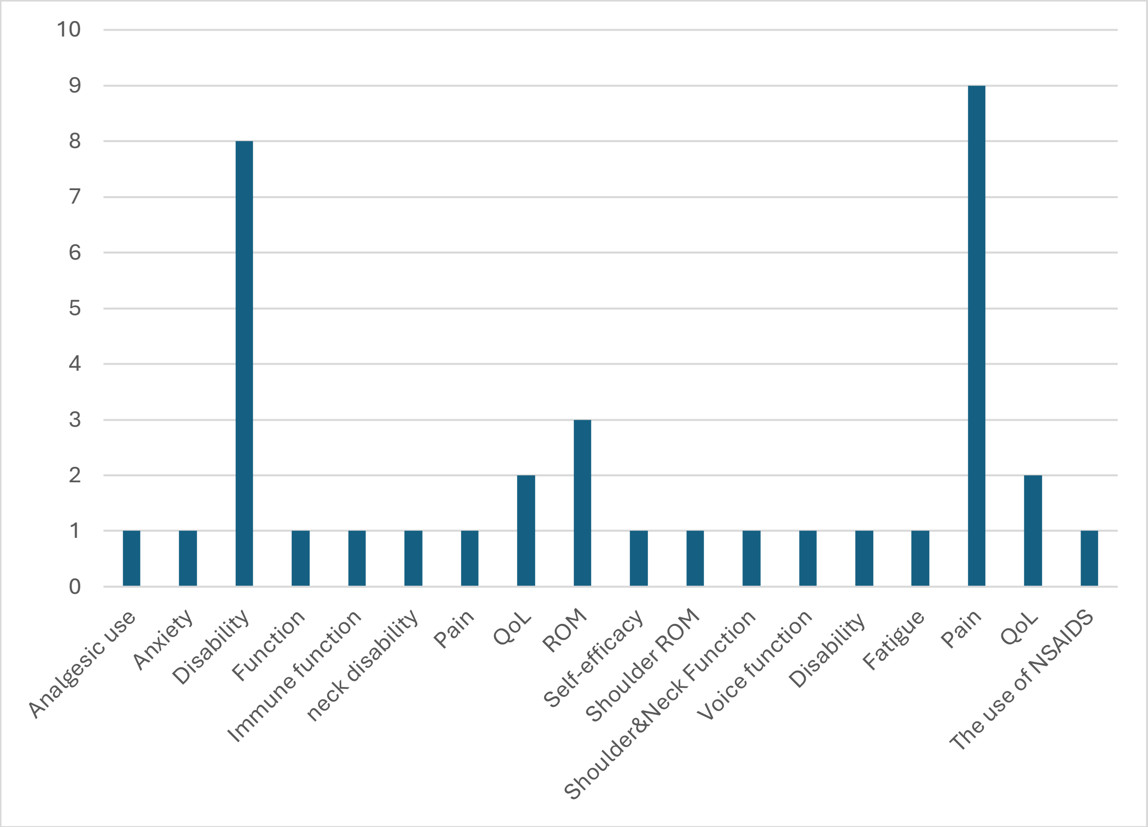

Supplementary Figure S3: Frequency of reported outcome domains across included studies (pivot chart – shows the frequency with which pain, disability, cervical range of motion, quality of life, and other postoperative recovery domains were evaluated).
