# Supplementary material for: Postoperative Rehabilitation After Thyroidectomy: A Scoping Review of Stretching, Manual Therapy, and Kinesio Taping Interventions
Source: J Clin Med. 2025 Dec 24;15(1):132. doi: 10.3390/jcm15010132 (PMC12787179; doi:10.3390/jcm15010132)
Supplement: Supplementary file 1 [file jcm-15-00132-s001.zip › Supplementary Table S1.pdf]

Supplementary Table S1. Full Electronic Search Strategy. Complete search strategies used for PubMed, Scopus, Embase, Web of Science and the Cochrane Library. Searches were conducted in August 2025 and were limited to studies published betweend January 2000 and March 2025.

Database	Search Strategy	Filters/Limits
PubMed	(thyroidectomy[MeSH Terms] OR thyroidectomy[tiab] OR "thyroid surgery"[tiab] OR "thyroid gland/surgery"[MeSH Terms]) AND (rehabilitation[MeSH Terms] OR rehabilitation[tiab] OR physiotherapy[tiab] OR "physical therapy"[tiab] OR kinesiotherapy[tiab] OR "kinesio taping"[tiab] OR exercise[tiab] OR stretching[tiab]) AND (postoperative[tiab] OR postsurgical[tiab] OR "post-surgical"[tiab] OR recovery[tiab])	Humans, Adults, English, 2000-2025
Scopus	TITLE-ABS-KEY (thyroidectomy OR "thyroid surgery") AND TITLE-ABS-KEY (rehabilitation OR physiotherapy OR "physical therapy" OR stretching OR exercise OR kinesiotherapy OR "kinesio taping") AND TITLE-ABS-KEY (postoperative OR postsurgical OR recovery)	English, 2000-2025
Embase	'thyroidectomy'/exp OR thyroidectomy:ti,ab OR "thyroid surgery":ti,ab AND ('rehabilitation'/exp OR rehabilitation:ti,ab OR physiotherapy:ti,ab OR "physical therapy":ti,ab OR exercise:ti,ab OR stretching:ti,ab OR kinesiotherapy:ti,ab OR "kinesio taping":ti,ab) AND (postoperative:ti,ab OR postsurgical:ti,ab OR recovery:ti,ab)	English, Adults, Humans, 2000-2025
Web of Science	TS = (thyroidectomy OR "thyroid surgery") AND TS = (rehabilitation OR physiotherapy OR "physical therapy" OR stretching OR exercise OR kinesiotherapy OR "kinesio taping") AND TS = (postoperative OR postsurgical OR recovery)	English, 2000-2025
Cochrane Library	(thyroidectomy OR "thyroid surgery") AND (rehabilitation OR physiotherapy OR "physical therapy" OR stretching OR exercise OR kinesiotaping)	Trials filter, English
